# Clinical Performance of REAC-Based ACT, CO, and MO-IBZ Protocols in Routine Practice: A Prospective Real-World Observational PMCF Study

**DOI:** 10.3390/jcm15052048

**Published:** 2026-03-07

**Authors:** Vania Fontani, Arianna Rinaldi, Bruna Lombardi, Salvatore Rinaldi

**Affiliations:** 1 Department of Reparative and Regenerative Medicine, Rinaldi Fontani Institute, 50144 Florence, Italy; vfontani@irf.it (V.F.); ari@irf.it (A.R.); 2 Department of Adaptive Neuro Psycho Physio Pathology and Neuro Psycho Physical Optimization, Rinaldi Fontani Institute, 50144 Florence, Italy; 3 Research Department, Rinaldi Fontani Foundation, 50144 Florence, Italy; brunalombardi54@gmail.com

**Keywords:** post-market clinical follow-up, real-world evidence, patient-reported outcomes, chronic low-grade inflammation, pain, sleep disturbance, fatigue, biomodulation

## Abstract

**Background/Objectives:** Chronic low-grade inflammation underlies persistent pain, sleep disturbance, fatigue, and reduced perceived well-being. ACT (anti-inflammatory cellular treatment), CO (circulatory optimization), and MO (metabolic optimization) are non-invasive REAC-based biomodulation protocols within the Inside Blue Zone (IBZ) framework, yet real-world evidence on patient-reported outcomes remains limited. The aim of this study was to evaluate pain intensity and symptom burden (sleep disturbance, fatigue, perceived well-being) in subjects undergoing ACT, CO, and MO within a Post-Market Clinical Follow-Up (PMCF) framework. **Methods:** This prospective observational PMCF study enrolled 50 subjects receiving sequential ACT, CO, and MO in routine practice. Pain was assessed at baseline (T0), end of treatment (T1), and follow-up (T2) using a Visual Analog Scale (VAS). Secondary outcomes were analyzed through clinically meaningful severity categories. **Results:** VAS scores decreased significantly from T0 to T1 (t(49) = 21.37, *p* < 0.001, Cohen’s d = 3.02) and remained reduced at T2. Seventy-eight percent met responder criteria. Secondary outcomes shifted toward lower severity categories at both timepoints. No adverse events occurred. **Conclusions:** Sequential ACT, CO, and MO produced clinically meaningful pain reductions and favorable symptom severity shifts with good tolerability, supporting clinical performance of this REAC-based approach in chronic low-grade inflammatory conditions.

## 1. Introduction

Chronic low-grade inflammation is increasingly recognized as a common biological background underlying a wide range of persistent clinical symptoms, including chronic pain [1], sleep disturbances [2], fatigue, and progressive impairment in quality of life [3,4]. Unlike acute inflammatory conditions, this form of inflammation often develops in the absence of overt laboratory abnormalities and evolves silently over time. As a result, affected individuals may experience substantial functional burden despite the lack of clearly identifiable biomarkers, making both diagnosis and treatment particularly challenging.

Current research suggests that chronic low-grade inflammation is not solely driven by classical immune mechanisms, but rather reflects a broader state of dysfunctional adaptation [5] involving inflammatory regulation, tissue perfusion, metabolic balance, and neurophysiological control [6]. In this context, increasing attention has been directed toward the role of endogenous bioelectrical activity as a coordinating factor in cellular and systemic homeostasis [7,8]. Alterations in bioelectrical signaling have been proposed to contribute to impaired regulation of inflammatory pathways, microcirculatory dysfunction, and metabolic dysregulation, thereby sustaining chronic symptomatology [9].

Within this conceptual framework, different therapeutic hypotheses have emerged. Conventional approaches largely focus on symptom suppression or pathway-specific modulation, often yielding incomplete or transient benefits in complex, multifactorial conditions. In contrast, emerging biomodulation strategies aim to support restoration of adaptive regulatory processes by acting on foundational physiological mechanisms rather than isolated symptoms. However, real-world clinical evidence supporting these approaches, particularly in routine practice settings, remains limited and heterogeneous.

Radio Electric Asymmetric Conveyer (REAC) technology is a non-invasive bioelectrical modulation approach designed to interact with endogenous bioelectrical activity involved in physiological regulation. Unlike conventional electrostimulation techniques aimed at inducing direct neural or muscular activation, REAC-based treatments are not intended to produce immediate symptomatic or analgesic effects. Rather, they are hypothesized to support adaptive regulatory processes by influencing endogenous bioelectrical signaling related to inflammatory modulation, tissue perfusion, and metabolic balance.

Anti-inflammatory cellular treatment (ACT), circulatory optimization (CO), and metabolic optimization (MO) are non-invasive REAC-based biomodulation protocols delivered within the Inside Blue Zone (IBZ) framework and designed to act sequentially on key domains implicated in chronic low-grade inflammatory states [10] and adaptive dysregulation.

ACT primarily targets the modulation of inflammatory responses, including chronic and subclinical inflammatory processes. CO is focused on improving tissue perfusion and microcirculatory dynamics, thereby supporting oxygen and nutrient delivery at the tissue level. MO addresses metabolic dysregulation, contributing to the progressive restoration of functional balance at both cellular and systemic levels. The sequential application of these protocols reflects a conceptual model aimed at the restoration of adaptive homeostasis, rather than isolated symptom control.

In routine clinical practice, patients undergoing ACT, CO, and MO protocols within the IBZ framework frequently report improvements in pain perception, sleep disturbance, fatigue, and overall well-being. However, systematic evaluation of these multidimensional patient-reported outcomes using validated instruments within a structured post-market clinical follow-up (PMCF) framework remains limited.

PMCF studies [11] are specifically designed to address this gap by generating real-world evidence on clinical performance and tolerability under conditions of normal use [12,13].

The present study was therefore designed as a prospective, observational PMCF investigation to evaluate changes in pain, sleep disturbance, fatigue, and health-related quality of life in subjects undergoing ACT, CO, and MO-IBZ protocols in routine clinical practice. By focusing on patient-reported outcome domains commonly assessed in the literature [14,15,16,17] and predefined follow-up intervals, this study aims to provide structured real-world evidence supporting the clinical performance of this sequential biomodulation approach in conditions associated with chronic low-grade inflammation.

## 2. Materials and Methods

### 2.1. Study Design

This study was designed as a prospective, observational investigation conducted within a PMCF framework [12]. Data were collected prospectively at predefined timepoints during routine care and subsequently anonymized prior to analysis. All participants were treated in routine clinical practice according to a predefined sequence of REAC ACT, CO, and MO-IBZ protocols. The study aimed to collect real-world data on clinical performance and tolerability under conditions of normal use.

The study was conducted in accordance with applicable post-market surveillance and clinical follow-up requirements for medical devices. All treatments were delivered using a certified medical device based on Radio Electric Asymmetric Conveyer (REAC) technology (BENE mod. 110, ASMED, Scandicci, Italy), according to manufacturer-defined protocols.

As this study was conducted in a routine clinical practice setting, participants were allowed to continue their usual pharmacological treatments. No protocol-driven modifications of ongoing therapies were required or implemented during the treatment cycle. Concomitant medications, including analgesics or anti-inflammatory drugs, were not standardized and were managed according to usual care. Therefore, the collected outcomes reflect real-world clinical conditions rather than controlled experimental settings.

#### Participant Recruitment and Consent

Participants were recruited among adult subjects undergoing routine clinical evaluation and treatment at the Rinaldi Fontani Institute. Eligibility for treatment was determined exclusively on clinical grounds, independent of study participation.

All subjects were informed that participation in the observational PMCF study was voluntary and that refusal to participate would not affect access to treatment or standard clinical care. Written informed consent for the use of anonymized clinical and patient-reported data for research purposes was obtained from all participants prior to data inclusion.

To minimize the risk of undue influence, no financial incentives were provided for study participation, and outcome data were collected as part of routine clinical follow-up rather than through protocol-driven interventions.

### 2.2. Outcomes

The primary outcome was the change in pain intensity measured using a 10-cm Visual Analog Scale (VAS; range 0–10), with higher scores indicating greater pain severity [14]. Pain assessments were performed at baseline (T0), at the end of the treatment cycle (T1), and at follow-up (T2) 4–8 weeks post-treatment.

For secondary outcomes [18], symptom severity was evaluated using internally derived severity categories based on symptom domains, rather than instrument-specific numerical scores, to support clinically meaningful interpretation in a real-world PMCF setting.

All outcome measures were administered at T0, T1, and T2. Secondary symptom ratings were collected using internally developed questionnaires (Italian version) and analyzed as severity categories.

Outcomes were selected to capture clinically relevant domains commonly associated with chronic low-grade inflammatory conditions, including pain perception, sleep regulation, fatigue, and perceived well-being.

Internally developed questionnaires included simple patient-reported items addressing perceived sleep quality, daytime fatigue, and overall well-being, rated on ordinal severity scales and subsequently categorized for clinical interpretability.

### 2.3. Scoring and Interpretation

For the primary outcome, a reduction of at least 30% in VAS score from baseline was predefined as a clinically meaningful improvement. Although standardized patient-reported instruments were available at the time of data collection, secondary outcomes were analyzed and reported using internally derived symptom severity categories to support clinically meaningful interpretation in a real-world PMCF context [12,17].

### 2.4. Statistical Analysis

Descriptive statistics were used to summarize baseline characteristics and outcome measures at each timepoint. Continuous variables were reported as mean ± standard deviation or as median and interquartile range, as appropriate. Categorical variables were summarized as counts and percentages.

The primary analysis evaluated within-subject changes in pain intensity (VAS) from baseline (T0) to end of treatment (T1). Normality of within-subject differences was assessed using distribution-based criteria; when assumptions were not met, non-parametric equivalents yielded convergent results. Paired comparisons were performed using two-tailed paired t-tests or non-parametric equivalents when normality assumptions were not met. Changes from baseline to follow-up (T2) were analyzed using the same approach to assess durability of effects. Effect sizes for within-subject changes were calculated to support interpretation of clinical relevance.

Secondary outcomes related to sleep disturbance, fatigue, and perceived well-being were evaluated using paired within-subject comparisons based on internally derived symptom severity categories. Severity categories were defined using baseline score quartiles; therefore, baseline distributions are by definition evenly balanced across severity levels. Secondary outcome analyses were considered supportive and exploratory, and results were primarily interpreted in terms of changes in symptom severity distribution over time. No formal adjustment for multiple comparisons was applied.

Responder analyses were conducted for the primary endpoint, defining responders as participants achieving a ≥30% reduction in VAS score from baseline.

Analyses were performed on a complete-case basis for each endpoint, including participants with available paired assessments at the relevant timepoints. Sensitivity analyses compared baseline-to-end-of-treatment (T0–T1) and baseline-to-follow-up (T0–T2) results to assess the impact of missing follow-up data. Statistical significance was set at a two-sided *p* value < 0.05 for the primary endpoint. All analyses were conducted using standard statistical software (IBM SPSS Statitistics 22).

Given the PMCF and real-world observational nature of the study, statistical analyses were intentionally focused on within-subject change estimation and clinical interpretability rather than on causal inference. Effect sizes were therefore reported to complement *p*-values and support clinical relevance independently of sample size.

### 2.5. Bias Mitigation and Data Integrity

Given the observational nature of the study and the involvement of the treating institution, several measures were adopted to mitigate potential sources of bias. Pain intensity was assessed using a widely accepted validated instrument (VAS), while secondary symptom domains were evaluated using predefined severity categories; all outcomes were patient-reported, collected systematically at predefined timepoints, and analyzed on anonymized datasets.

Outcome assessment was not modified based on treatment response, and no interim analyses or outcome-driven protocol modifications were performed. The study followed a predefined PMCF framework, and all available paired data were included in the analyses to reduce selective reporting.

## 3. Results

### 3.1. Study Population

A total of 50 participants were enrolled in the study and completed the full treatment cycle, providing evaluable paired data for the primary endpoint at baseline (T0) and end of treatment (T1). Follow-up data at T2 were available for all participants. Baseline demographic and clinical characteristics are summarized in Table 1. Sex distribution reflects the characteristics of the population treated in routine clinical practice and was not constrained by predefined enrollment quotas. All participants were adults, with ages ranging from 32 to 76 years.

### 3.2. Primary Outcome: Pain Intensity

A statistically significant reduction in pain intensity, measured by the Visual Analog Scale (VAS), was observed following treatment. Mean VAS scores decreased from 6.77 ± 0.94 at baseline (T0) to 4.2 ± 1.0 at the end of the treatment cycle (T1) (t(49) = 21.37, *p* < 0.001, Cohen’s d = 3.02).

**Figure 1:**
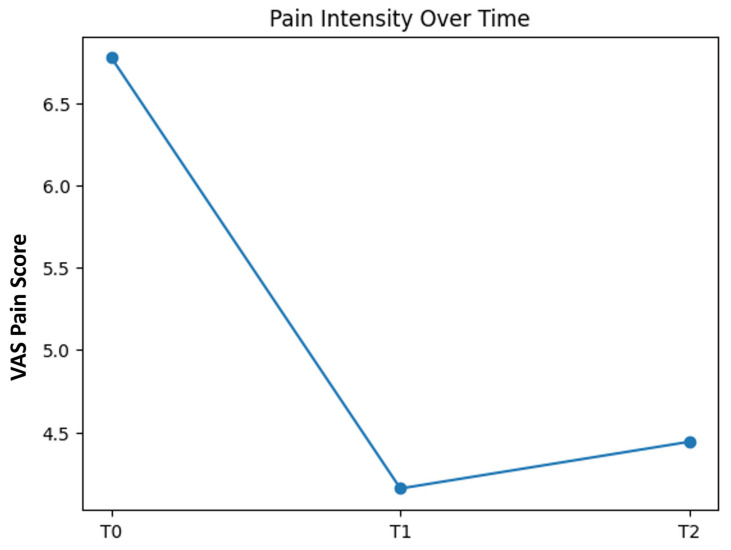
Pain course. Mean pain intensity (VAS) at baseline (T0), end of treatment cycle (T1), and follow-up (T2).

At follow-up (T2), pain reduction remained significant compared with baseline, with mean VAS scores of 4.4 ± 1.2 (t(49) = 18.89, *p* < 0.001, and Cohen’s d = 2.67 vs. T0), indicating maintenance of the treatment effect over time. The time course of pain intensity across assessment points is shown in Figure 1.

### 3.3. Responder Analysis

Responder analysis was included to provide a clinically oriented real-world perspective, in which the proportion of patients achieving a predefined meaningful improvement is often more informative than changes in group means alone.

**Figure 2:**
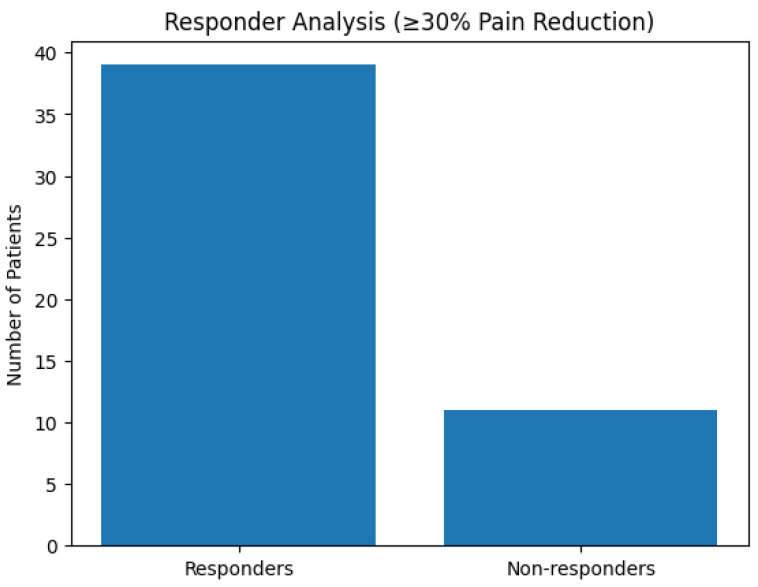
Responder analysis. Number of responders and non-responders at the end of the treatment cycle (T1), based on a predefined responder criterion of ≥30% reduction in VAS pain score from baseline.

Using the predefined responder criterion (≥30% reduction in VAS score from baseline), 39 participants (78%) were classified as responders at the end of the treatment cycle (T1). Responder status was largely maintained at follow-up, supporting the clinical relevance and durability of pain improvement. Responder classification reflects predefined measurement thresholds and does not imply absence of clinical benefit in participants who did not meet responder criteria. The distribution of responders and non-responders is illustrated in Figure 2.

### 3.4. Secondary Outcomes

#### 3.4.1. Sleep Disturbance

At baseline (T0), participants presented a broad distribution of sleep disturbance severity, reflecting the distribution-based definition of severity thresholds. A substantial proportion of participants reported moderate to severe sleep disturbance at baseline.

Following completion of the treatment cycle (T1), a clear shift toward lower severity categories was observed. The proportion of subjects classified as having moderate or severe sleep disturbance decreased substantially, while the proportion of those reporting absent or mild sleep disturbance increased. This shift was consistent across the study population and reflected an overall improvement in subjective sleep disturbance.

At follow-up (T2), improvements in sleep disturbance were largely maintained, with severity distributions remaining markedly more favorable than at baseline. Although minor fluctuations were observed in individual subjects, the overall pattern suggested durability of the observed sleep-related benefits.

**Figure 3:**
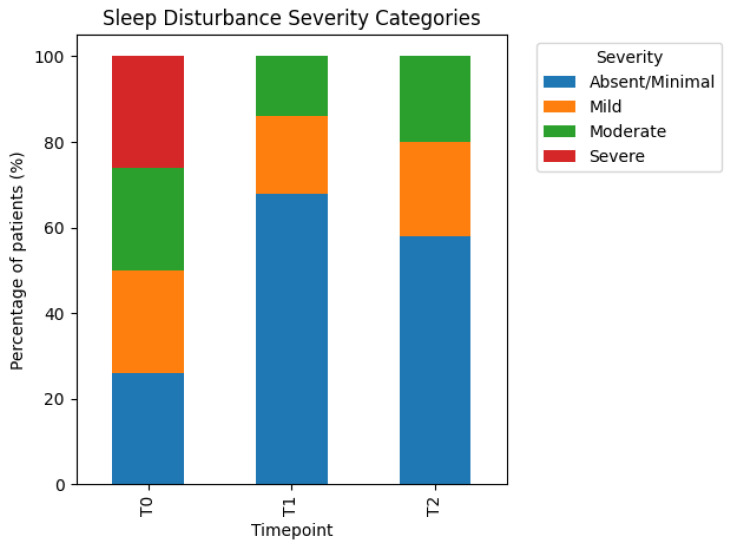
Sleep disturbance severity shifts. Distribution of sleep disturbance severity categories at baseline (T0), end of treatment cycle (T1), and follow-up (T2). Stacked bars represent the percentage of patients classified as having absent/minimal, mild, moderate, or severe sleep disturbance at each assessment timepoint. Severity categories were derived from internally collected symptom rating questionnaires and defined using baseline score distributions, then applied unchanged to post-treatment and follow-up assessments.

These findings indicate that the treatment sequence was associated with a clinically meaningful reduction in sleep-related symptom burden over time (Figure 3).

#### 3.4.2. Fatigue

At baseline (T0), participants exhibited a broad distribution of fatigue severity, reflecting the distribution-based definition of severity thresholds. Moderate and severe fatigue categories were well represented at baseline.

At the end of the treatment cycle (T1), a pronounced reduction in fatigue severity was observed. A substantial proportion of participants shifted from moderate or severe fatigue to mild or absent fatigue categories, indicating a clinically relevant improvement in perceived energy levels and functional capacity.

At follow-up (T2), the majority of participants maintained the improvements achieved at T1. The distribution of fatigue severity categories remained substantially improved compared with baseline, suggesting that treatment-related effects on fatigue were sustained beyond the active treatment phase.

**Figure 4:**
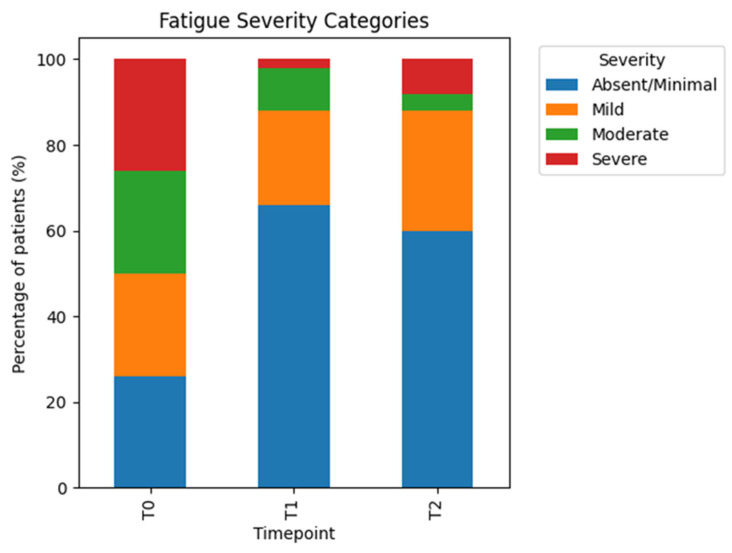
Fatigue severity shifts. Distribution of fatigue severity categories at baseline (T0), end of treatment cycle (T1), and follow-up (T2). Stacked bars show the percentage of patients within each fatigue severity category (absent/minimal, mild, moderate, severe). Severity thresholds were defined at baseline using internally collected symptom ratings and consistently applied across subsequent assessments to visualize changes in fatigue burden over time.

Overall, the observed fatigue severity shifts support a favorable effect of the treatment sequence on this key symptom domain (Figure 4).

#### 3.4.3. Perceived Well-Being

At baseline (T0), perceived well-being impairment showed a broad distribution across severity categories, consistent with the distribution-based definition of thresholds. Categories reflecting moderate and marked impairment were well represented at baseline.

Following treatment completion (T1), a clinically relevant improvement in perceived well-being was observed. A marked shift toward lower levels of well-being impairment was evident, with a growing proportion of participants reporting minimal or mild impairment.

At follow-up (T2), improvements in perceived well-being were largely preserved, indicating sustained benefits in overall health perception and daily functioning. While individual variability was present, the group-level distribution continued to favor lower impairment categories relative to baseline.

**Figure 5:**
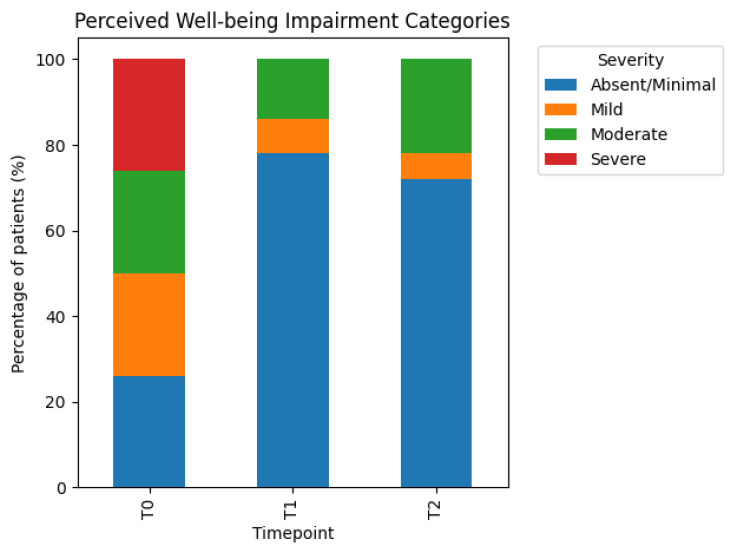
Perceived well-being impairment. Distribution of perceived well-being impairment severity categories at baseline (T0), end of treatment cycle (T1), and follow-up (T2). Stacked bars represent the proportion of patients classified as having minimal, mild, moderate, or marked impairment at each assessment timepoint. Severity categories were derived from internally collected patient-reported symptom ratings and applied longitudinally to illustrate changes in perceived well-being over time.

These results suggest that the treatment sequence was associated not only with symptom-specific improvements but also with a broader enhancement of patients’ perceived well-being. Severity category shifts for perceived well-being across assessment timepoints are illustrated in Figure 5.

### 3.5. Safety and Tolerability

No adverse events related to the treatment protocols were reported during the study period. The interventions were well tolerated, and no participants discontinued treatment due to adverse effects.

### 3.6. Missing Data

No relevant missing data were observed for the primary endpoint. Analyses conducted on complete paired datasets yielded consistent results across all outcome measures.

## 4. Discussion

In this prospective real-world observational study conducted within a PMCF framework, sequential ACT, CO, and MO-IBZ protocols were associated with clinically meaningful improvements in pain, sleep disturbance, fatigue, and health-related quality of life. Improvements were observed at the end of the treatment cycle and were largely maintained at follow-up, supporting both the clinical performance and durability of effects under routine practice conditions.

An important aspect in interpreting the observed pain reduction relates to the clinical characteristics of the study population. The pain reported by participants was not predominantly nociceptive or localized, but rather diffuse, often migratory, and typically poorly responsive to conventional therapeutic approaches, as reflected in routine clinical anamnesis. This type of pain is commonly associated with chronic low-grade inflammatory states and maladaptive regulatory processes, frequently occurring in the absence of overt structural lesions or laboratory abnormalities. In this context, the observed and sustained reduction in pain intensity should not be interpreted as a purely symptomatic analgesic effect, but rather as a clinical expression of a broader modulation of underlying adaptive and inflammatory dysregulation. The durability of pain improvement at follow-up further supports this interpretation, as diffuse inflammation-related pain is generally characterized by limited and transient responsiveness to standard symptom-oriented interventions.

A distinctive strength of the present study lies in the pragmatic approach adopted for the analysis and presentation of patient-reported outcomes [19,20] beyond pain.

Severity category-based reporting was deliberately adopted to enhance clinical interpretability in a real-world PMCF context. In routine practice, shifts from moderate–severe to mild or minimal symptom burden are often more clinically meaningful than marginal changes in numerical scores.

By defining severity thresholds at baseline and applying them unchanged across subsequent assessments, this approach allows transparent visualization of true symptom severity shifts over time.

Accordingly, secondary outcomes related to sleep disturbance, fatigue, and perceived well-being were evaluated using clinically meaningful severity categories derived from internally collected symptom ratings, emphasizing patient-centered outcomes that are directly relevant to everyday clinical decision-making and consistent with real-world evidence principles.

Beyond pain, improvements in sleep disturbance and fatigue highlight the broader impact of the treatment sequence on symptom domains that are closely intertwined with chronic inflammatory burden and adaptive dysregulation. Sleep and fatigue are increasingly recognized as key determinants of functional impairment and reduced quality of life in chronic inflammatory and stress-related conditions. Their concurrent improvement suggests a multidimensional clinical effect that extends beyond isolated analgesia, as further supported by consistent improvements in perceived well-being severity categories.

Taken together, these multidimensional improvements reflect clinically meaningful changes in domains that are central to health-related quality of life, rather than isolated symptom relief.

Previous clinical and experimental research has increasingly emphasized the role of dysregulated adaptive mechanisms, rather than isolated inflammatory pathways, in sustaining chronic low-grade inflammatory states. Emerging evidence suggests that altered endogenous bioelectrical activity may contribute to impaired coordination of inflammatory regulation, microcirculation, and metabolic processes. Within this context, REAC-based biomodulation has been proposed as a strategy to support adaptive regulatory processes rather than isolated symptom control. While the current study does not directly address mechanistic pathways, the observed pattern of patient-reported outcomes [20] aligns with prior observations that integrative, non-pathway-specific approaches may yield broader and more durable clinical benefits in complex chronic conditions.

It is noteworthy that not all participants met predefined responder criteria for pain reduction. This finding should not be interpreted as absence of treatment-related biological response. Rather, responder classification reflects measurement-based thresholds applied to a single symptom domain and may underestimate broader or multidimensional clinical benefits. In real-world settings, symptom perception and reporting may also be influenced by individual expectations, baseline symptom profiles, and the relative salience of different symptom domains, particularly when outcomes rely on patient-reported measures.

The sequential application of ACT, CO, and MO-IBZ protocols is conceptually aligned with the multifactorial nature of chronic low-grade inflammation. By addressing inflammatory modulation, tissue perfusion, and metabolic balance in a progressive manner, this approach may support restoration of adaptive homeostasis rather than transient symptom suppression. This conceptual model differs from conventional symptom-centered strategies and reflects a shift toward interventions aimed at rebalancing foundational regulatory processes.

From a broader clinical perspective, these findings contribute to the growing body of real-world evidence supporting the use of integrative, non-invasive biomodulation strategies in conditions characterized by chronic symptom burden and limited objective biomarkers.

In a broader health context, the symptom domains addressed in this study, pain regulation, sleep quality, fatigue, and perceived well-being, are also key determinants of functional resilience and healthy aging, particularly in populations exposed to chronic low-grade inflammatory burden.

Future research should aim to confirm these observations in controlled or comparative study designs, explore domain-specific responder profiles, and investigate the temporal dynamics of symptom improvement across different patient subgroups. Additional studies integrating objective physiological or biomolecular markers may further clarify the relationship between adaptive modulation and clinical outcomes, thereby refining patient selection and treatment sequencing.

This study has several limitations. Its observational design and lack of a control group preclude formal causal inference; therefore, the findings should be interpreted within the framework of real-world clinical evaluation rather than experimental efficacy testing. Nonetheless, when introduced in the context of otherwise stable therapeutic conditions, the REAC-based treatment sequence was associated with robust and clinically meaningful improvements that were maintained over time.

A further limitation of this study is the lack of a systematic stratification of concomitant pharmacological treatments. However, these therapies were already ongoing prior to study entry and were not modified during the treatment cycle, making them unlikely to explain the observed within-subject changes.

Nonetheless, the prospective design, predefined endpoints, use of patient-reported measures routinely employed in clinical practice, and structured follow-up mitigate some of these limitations and are fully consistent with the aims of PMCF investigations.

In conclusion, within a real-world PMCF setting, ACT, CO, and MO-IBZ protocols were associated with meaningful improvements across multiple patient-reported domains relevant to chronic low-grade inflammatory conditions, with good tolerability and sustained effects.

## 5. Conclusions

In a real-world PMCF setting, sequential ACT, CO, and MO-IBZ protocols were associated with clinically meaningful improvements in pain intensity and favorable shifts in symptom severity related to sleep disturbance, fatigue, and perceived well-being. Improvements were observed at the end of the treatment cycle and were largely maintained at follow-up, indicating durability of clinical effects under routine practice conditions.

By combining responder-based analysis for pain with severity category-based reporting for secondary symptom domains, this study provides a pragmatic and clinically interpretable representation of patient-reported outcomes [20] that is well suited to real-world and post-market evaluation. When introduced in a context of otherwise stable therapeutic conditions, the sequential REAC-based ACT, CO, and MO-IBZ protocols were associated with robust and clinically meaningful improvements that were maintained over time. Overall, the findings support the clinical performance, tolerability, and applicability of this REAC-based sequential biomodulation approach in patients with chronic symptom burden associated with low-grade inflammatory states.

## Figures and Tables

**Table 1:** Baseline demographic and clinical characteristics of the study population. Values are reported as mean ± standard deviation unless otherwise indicated. Sleep disturbance and fatigue outcomes are reported descriptively as severity categories and are therefore not summarized as numerical baseline values in this table. Predominant symptom pattern refers to the main symptom domain reported by participants at baseline.

Variable	Overall Population
Sex (female), *n* (%)	32 (64.0%)
Pain intensity (VAS, baseline), mean ± SD	6.77 ± 0.94
Pain responders at T1 (≥30% VAS reduction), *n* (%)	39 (78.0%)

## Data Availability

All relevant data generated or analyzed during this study are included in this published article.
